# Supplementary material for: Transcranial photobiomodulation improves insulin therapy in diabetic microglial reactivity and the brain drainage system
Source: Commun Biol. 2023 Dec 8;6:1239. doi: 10.1038/s42003-023-05630-3 (PMC10709608; doi:10.1038/s42003-023-05630-3)
Supplement: Supplementary file 2 — Supplementary Information [file 42003_2023_5630_MOESM2_ESM.pdf]

Supplementary Information

Transcranial photobiomodulation improves insulin therapy in diabetic microglial reactivity and the brain drainage system

Shaojun Liu[1,#], Dongyu Li[2,#], Tingting Yu[1], Jingtan Zhu[1], Oxana Semyachkina-Glushkovskaya[3,4], Dan Zhu[1,*]

[1] Britton Chance Center for Biomedical Photonics - MoE Key Laboratory for Biomedical Photonics, Wuhan National Laboratory for Optoelectronics - Advanced Biomedical Imaging Facility, Huazhong University of Science and Technology, 430074 Wuhan, Hubei, China.
[2] School of Optical Electronic Information - Advanced Biomedical Imaging Facility, Huazhong University of Science and Technology, 430074 Wuhan, Hubei, China.
[3] Saratov State University, Astrakhanskaya str. 83, 410012 Saratov, Russia.
[4] Physics Department, Humboldt University, Newtonstrasse 15, 12489 Berlin, Germany.
[#] These authors contributed equally to this work.

Corresponding author: Dan Zhu, E-mail: dawnzh@mail.hust.edu.cn

Supplementary Fig. 1 The changes of microglial branches accumulation at different distances from the injury in the CTR, DM-1W, DM-2W and DM-3W groups.

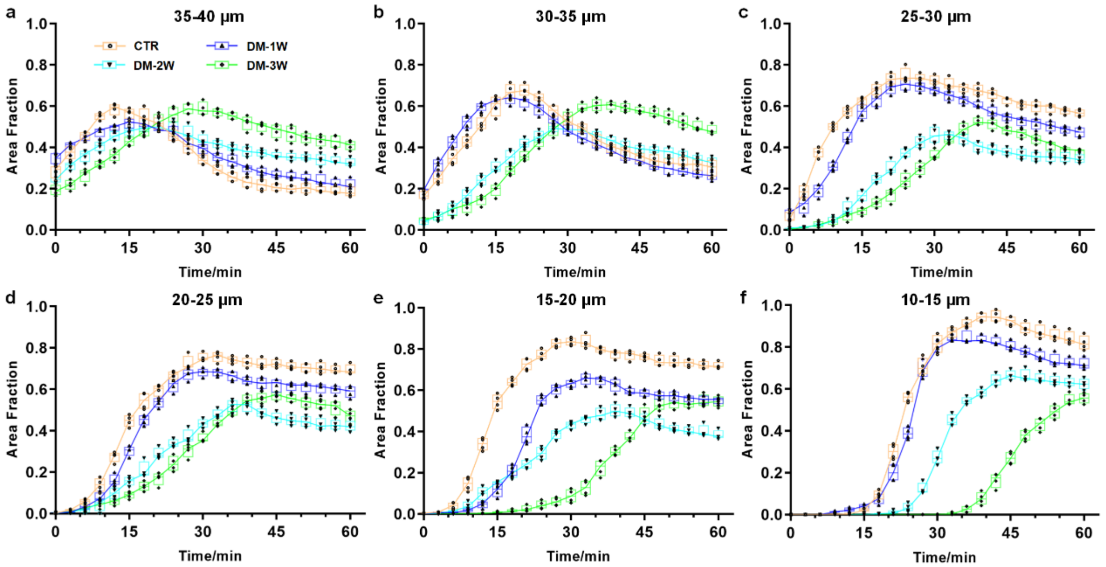

Changes of microglial branches accumulation at distances of 35-40 μm **(a)**, 30-35 μm **(b)**, 25-30 μm **(c)**, 20-25 μm **(d)**, 15-20 μm **(e)**, 10-15 μm **(f)** from the injury (the box indicates the upper and lower quantiles, the thick line in the box indicates the median and whiskers indicate 2.5th and 97.5th percentiles). Data were presented as mean ± standard deviation (n=4 mice in each group).

Supplementary Fig. 2 Design of tPBM treatment.

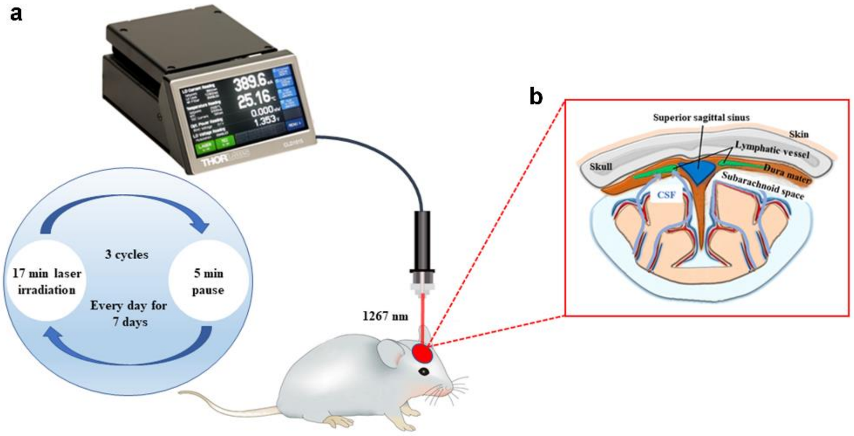

a The scheme of the 1267 nm laser irradiation (32 J/cm^2 laser dose). The 1267 nm laser irradiation was performed on the shaved head every day for one week using the sequence of 17 min irradiation, 5 min pause, 61 min in total. **b** Schematic diagram of the skull and brain structures below the laser irradiation site.

Supplementary Fig. 3 The changes of microglial characterization.

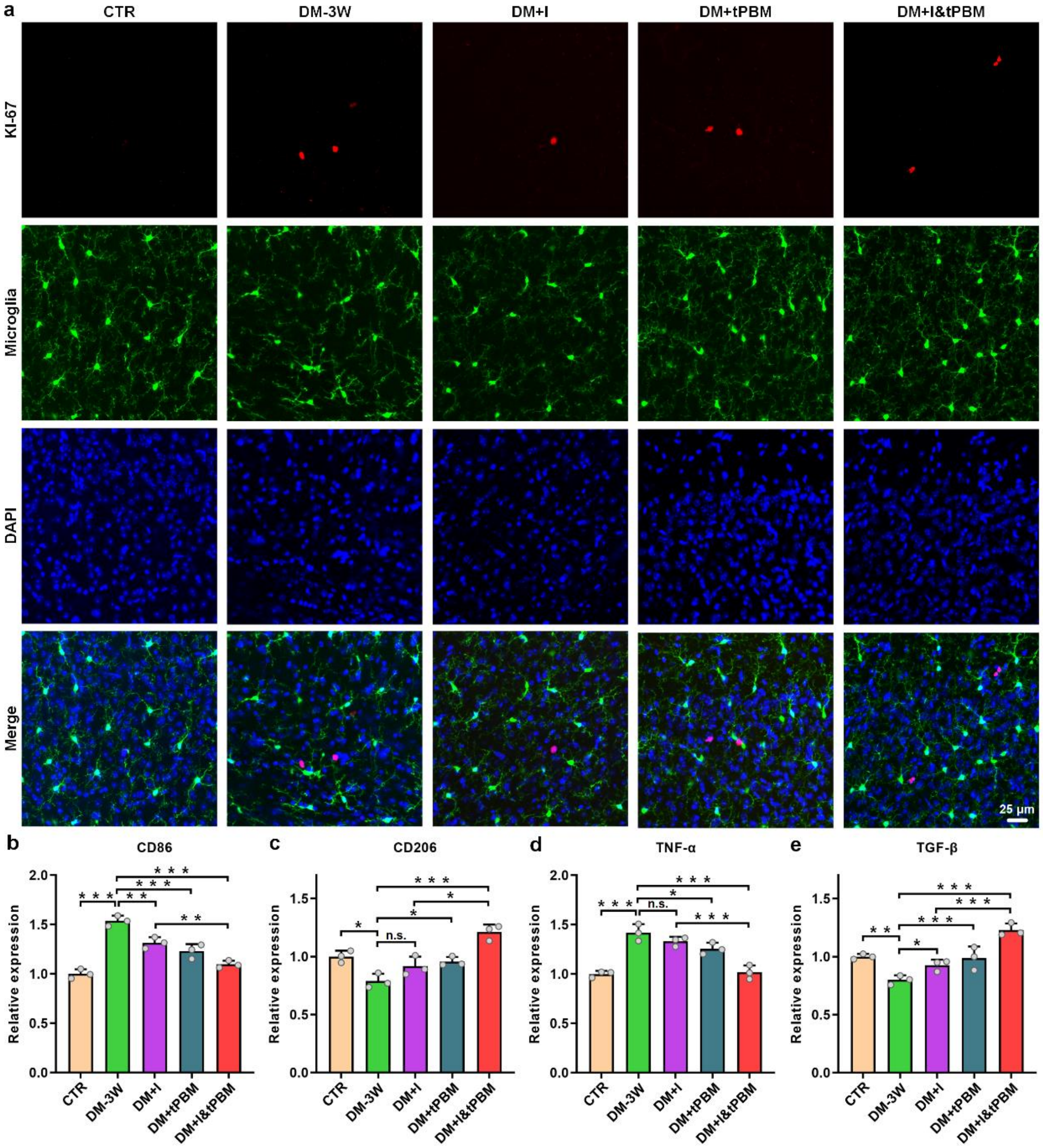

a Changes of microglial proliferation in the tested groups. **b-e** Changes of the expression of mRNA level of different microglia cytokines CD86 **(b)**, CD206 **(c)**, TNF-α **(d)**, TGF-β **(e)** in the tested groups**.** Data were presented as mean ± standard deviation (n=3 mice in each group), statistical significance **(b-e)** was assessed using one-way ANOVA, followed by Tukey's post hoc test. The statistical tests involved two-sided analyses (n.s., *, **, and *** indicate not significant, p<0.05, p<0.01 and p<0.001, respectively).

Supplementary Fig. 4 The changes of microglial branches accumulation at different distances from the injury in the CTR, DM-3W, DM+I, DM+tPBM and DM+I&tPBM groups.

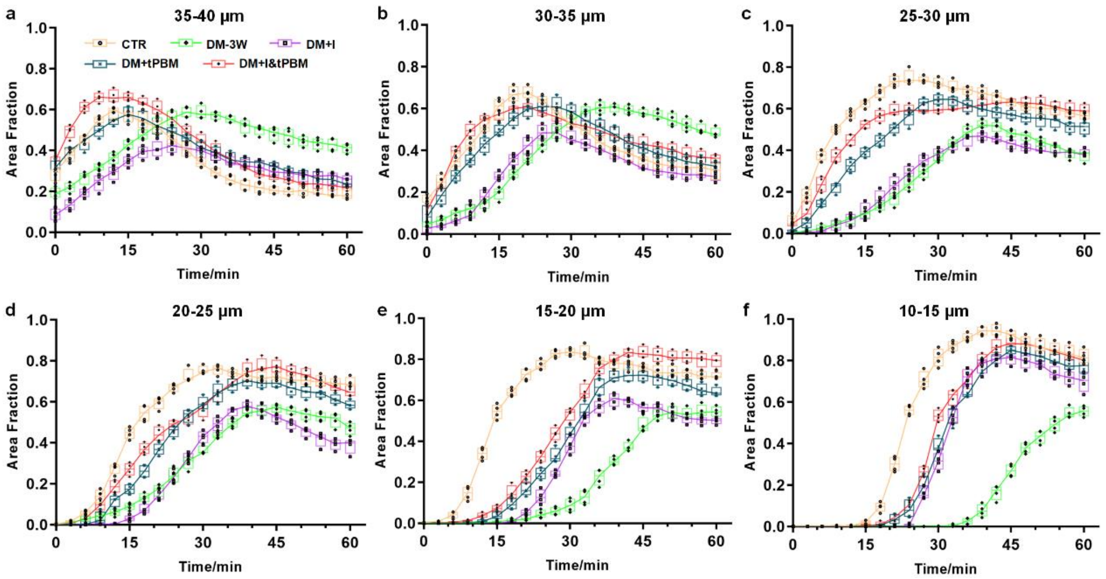

Changes of microglial branches accumulation at distances of 35-40 μm **(a)**, 30-35 μm **(b)**, 25-30 μm **(c)**, 20-25 μm **(d)**, 15-20 μm **(e)**, 10-15 μm **(f)** from the injury (the box indicates the upper and lower quantiles, the thick line in the box indicates the median and whiskers indicate 2.5th and 97.5th percentiles). Data were presented as mean ± standard deviation (n=4 mice in each group).

Supplementary Fig. 5 The influences of the 7 days course of tPBM on the density and the microglial morphology in healthy mice.

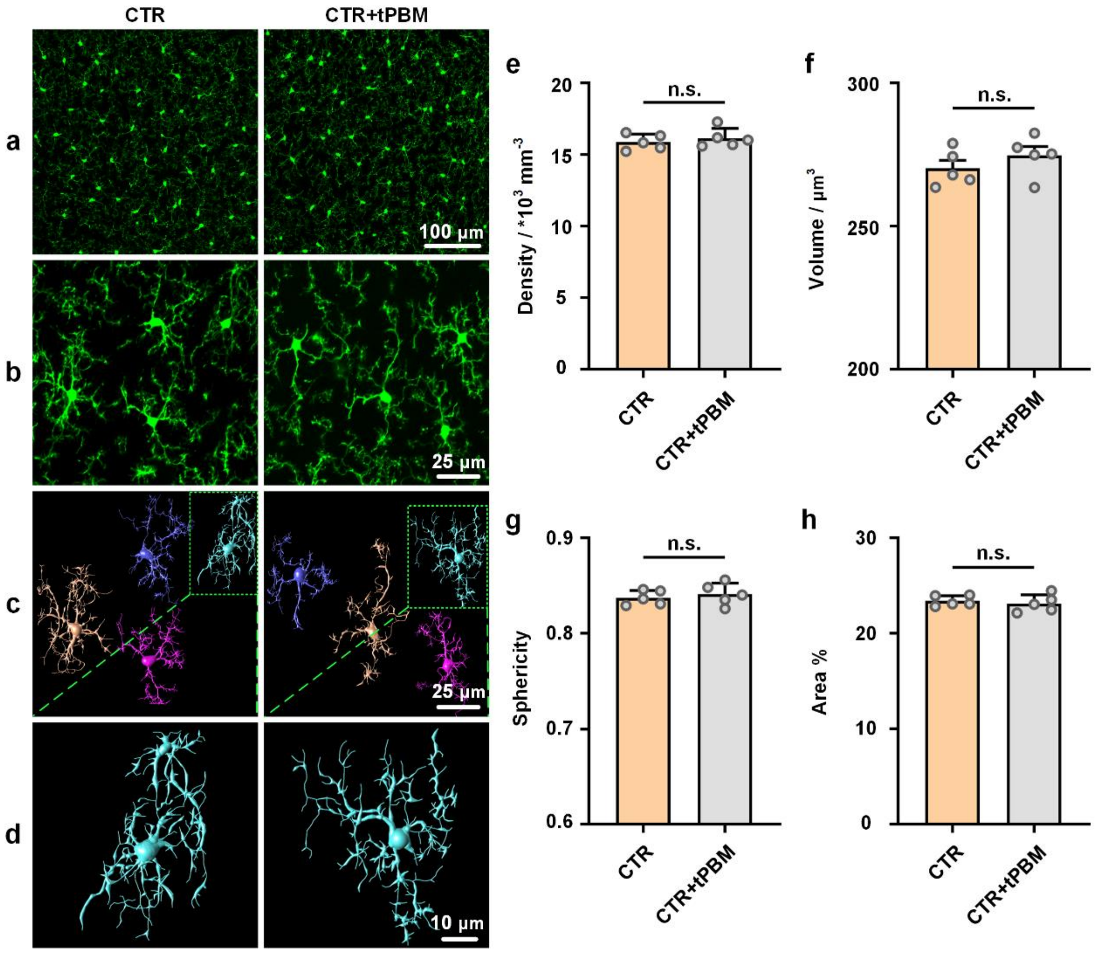

a Distribution of cortical microglia in the CTR and the CTR+tPBM groups. Scale bar: 100 μm. **b** Representative images of morphological changes of microglia in the groups of CTR and CTR+tPBM. Scale bar: 25 μm. **c** Three-dimensional reconstruction of microglia cells in the groups of CTR and CTR+tPBM. Scale bar: 25 μm. **d** Enlarged views of microglia in the green dashed box in **(c)**. Scale bar: 10 μm. **e-h** Quantification of changes in cortical microglial density **(e)**, soma volume **(f)**, soma sphericity **(g)**, and average percentage area of microglia **(h)**, respectively. Data were presented as mean ± standard deviation (n=5 mice in each group), statistical significance **(e-h)** was assessed using unpaired Student's t test. The statistical tests involved two-sided analyses (n.s. indicates not significant).

Supplementary Fig. 6 The influences of tPBM on the function of microglia in response to vascular injury in healthy mice.

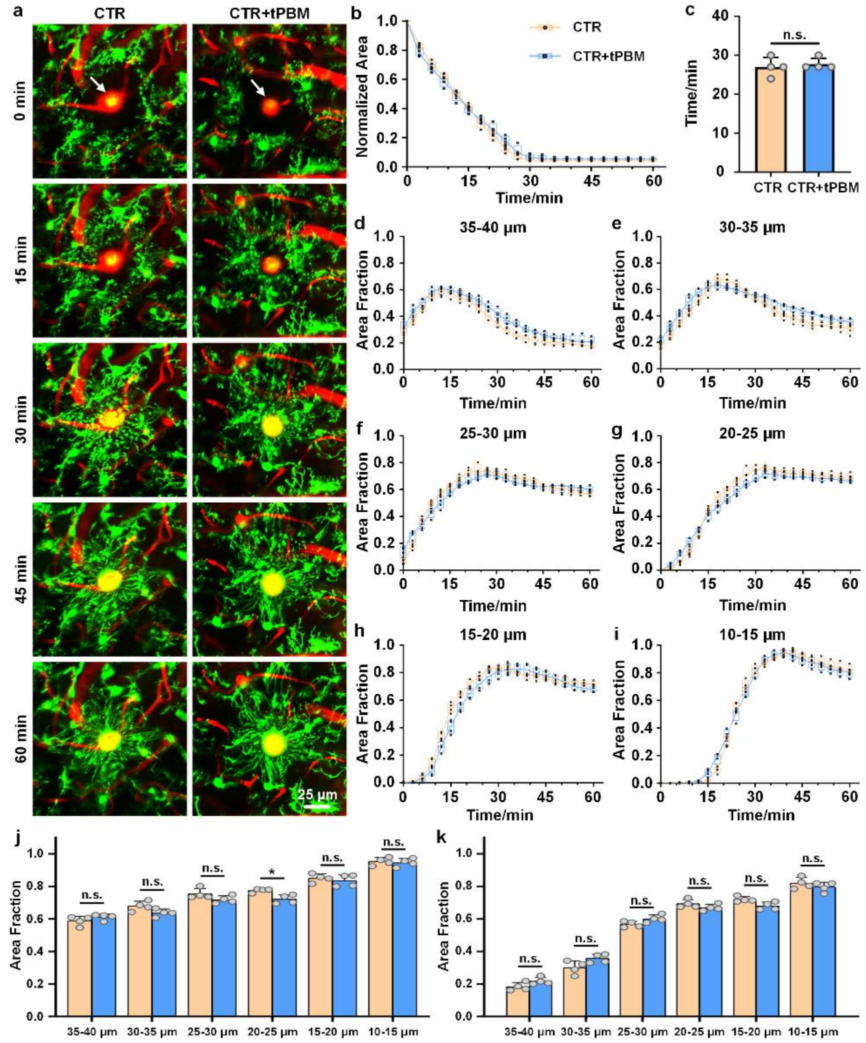

a Representative time series maximum intensity z projection images of microglia after vascular injury in the CTR and CTR+PBM groups. Scale bar: 25 µm. **b** The changes of polygon area bound by microglia processes closest to the injury in the tested groups (the box indicates the upper and lower quantiles, the thick line in the box indicates the median and whiskers indicate 2.5th and 97.5th percentiles). **c** Quantitative analysis of the time taken for the area of the polygon to be reduced to a minimum. **d-i** Graphs represent time series changes of microglia processes accumulation at the distances of 35-40 µm **(d)**, 30-35 µm **(e)**, 25-30 µm **(f)**, 20-25 µm **(g)**, 15-20 µm **(h)**, 10-15 µm **(i)** from the injury in the tested groups (the box indicates the upper and lower quantiles, the thick line in the box indicates the median and whiskers indicate 2.5th and 97.5th percentiles). **j** Quantification of the maximum percentage of microglial branches accumulation at different distances from the injury in the tested groups. **k** Quantification of the percentage of microglial branches accumulation at different distances 60 min after the injury in the tested groups. Data were presented as mean ± standard deviation (n=4 mice in each group), statistical significance **(c, j, k)** was assessed using unpaired Student's t test. The statistical tests involved two-sided analyses (n.s. indicates not significant).

Supplementary Fig. 7 Changes in cortex temperature induced by tPBM.

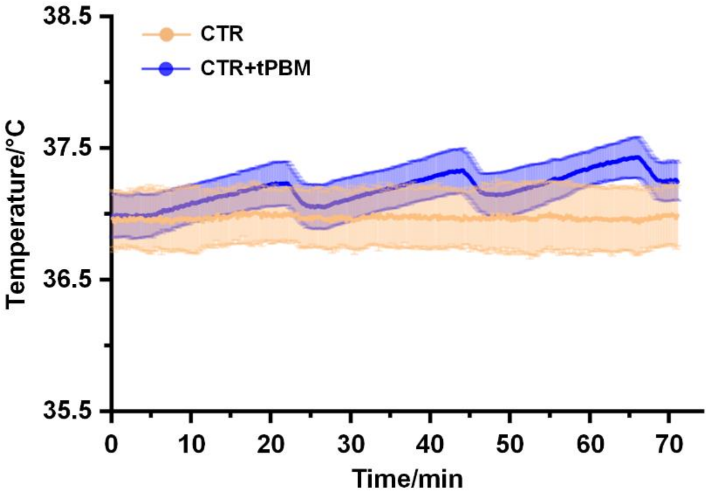

Data were presented as mean ± standard deviation (n=5 mice in each group).

Supplementary Fig. 8 The analysis of energy and locomotor activity changes in mice.

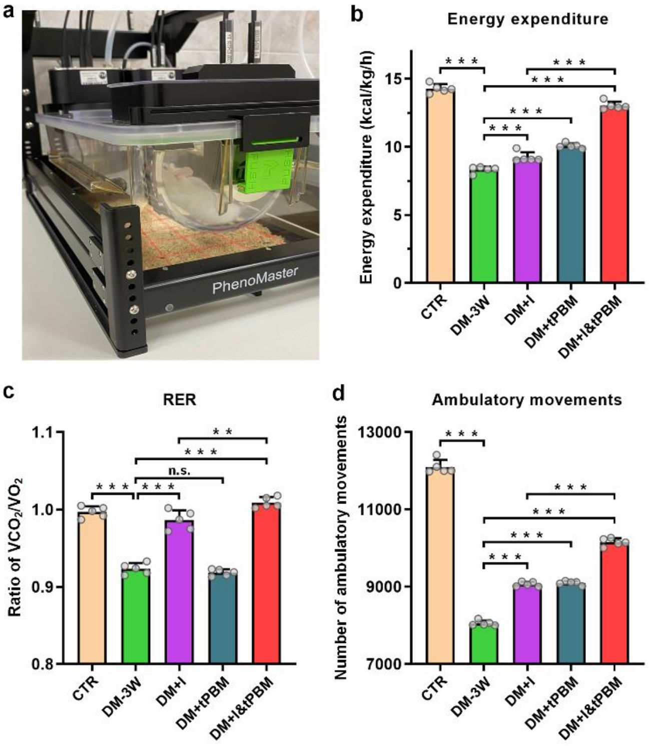

a The system for automatic recording of behavioral and metabolic parameters in mice (PhenoMaster TSE Systems). **b-d** Quantitative analysis of changes in energy expenditure **(b)**, respiratory exchange ratio (RER) **(c)** and locomotor activity **(d)** in mice. Data were presented as mean ± standard deviation (n=5 mice in each group), statistical significance **(b-d)** was assessed using one-way ANOVA, followed by Tukey's post hoc test. The statistical tests involved two-sided analyses (n.s., *, **, and *** indicate not significant, $p<0.05$, $p<0.01$ and $p<0.001$, respectively).

Supplementary Fig. 9 Visualization and quantitative analysis of BBB disruption at different stages of diabetes.

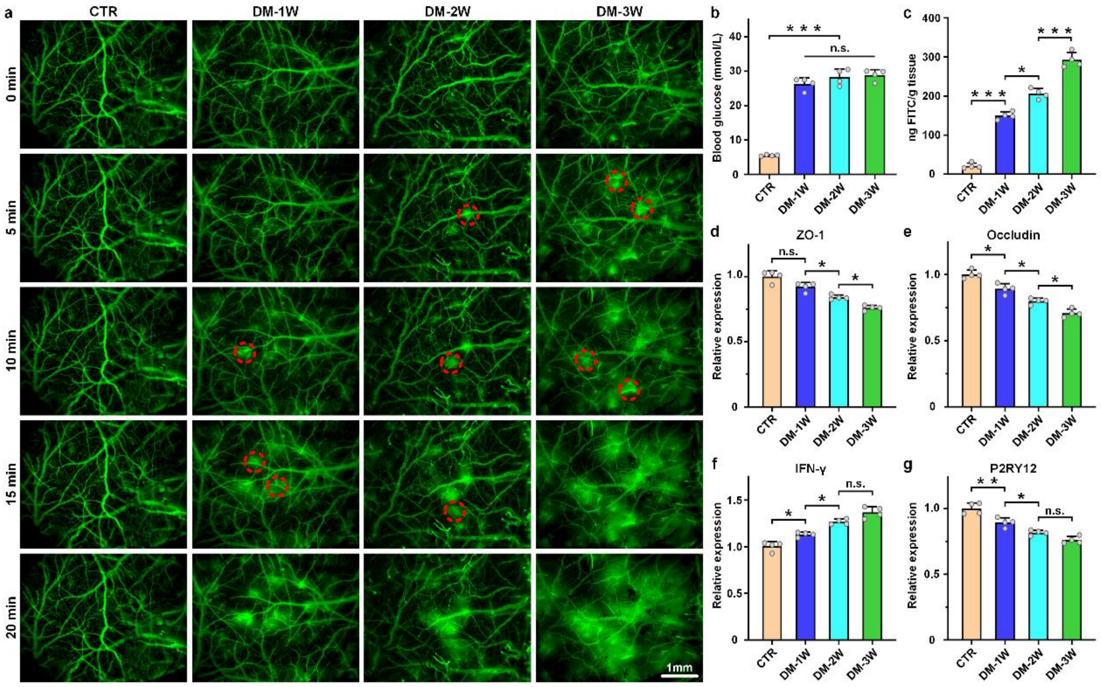

a Time series fluorescence imaging of cortical vasculature after 70kDa FITC-Dextran injection in mice of tested groups. The red circles indicate the locations of vascular leakage. Scale bar: 1 mm. **b** Continuous changes of blood glucose in mice after establishment of diabetes model. **c** Quantitative analysis the extravasation of FITC-Dextran in mice brain tissue at different stages of diabetes. **d, e** Changes of tight junction proteins ZO-1 **(d)** and Occludin **(e)** at different stages of diabetes, respectively. **f** Changes of proinflammatory cytokine IFN-γ expression in brain tissue at different stages of diabetes. **g** Changes in the expression of microglia chemokine receptor P2RY12 with the progression of diabetes. Data were presented as mean ± standard deviation (n=4 mice in each group), statistical significance **(b-g)** was assessed using repeated measurements one-way ANOVA. The statistical tests involved two-sided analyses (n.s., *, **, and *** indicate not significant, $p<0.05$, $p<0.01$, and $p<0.001$, respectively).

Supplementary Fig. 10 Effects of tPBM (7-day courses) on the diameter of the MLVs.

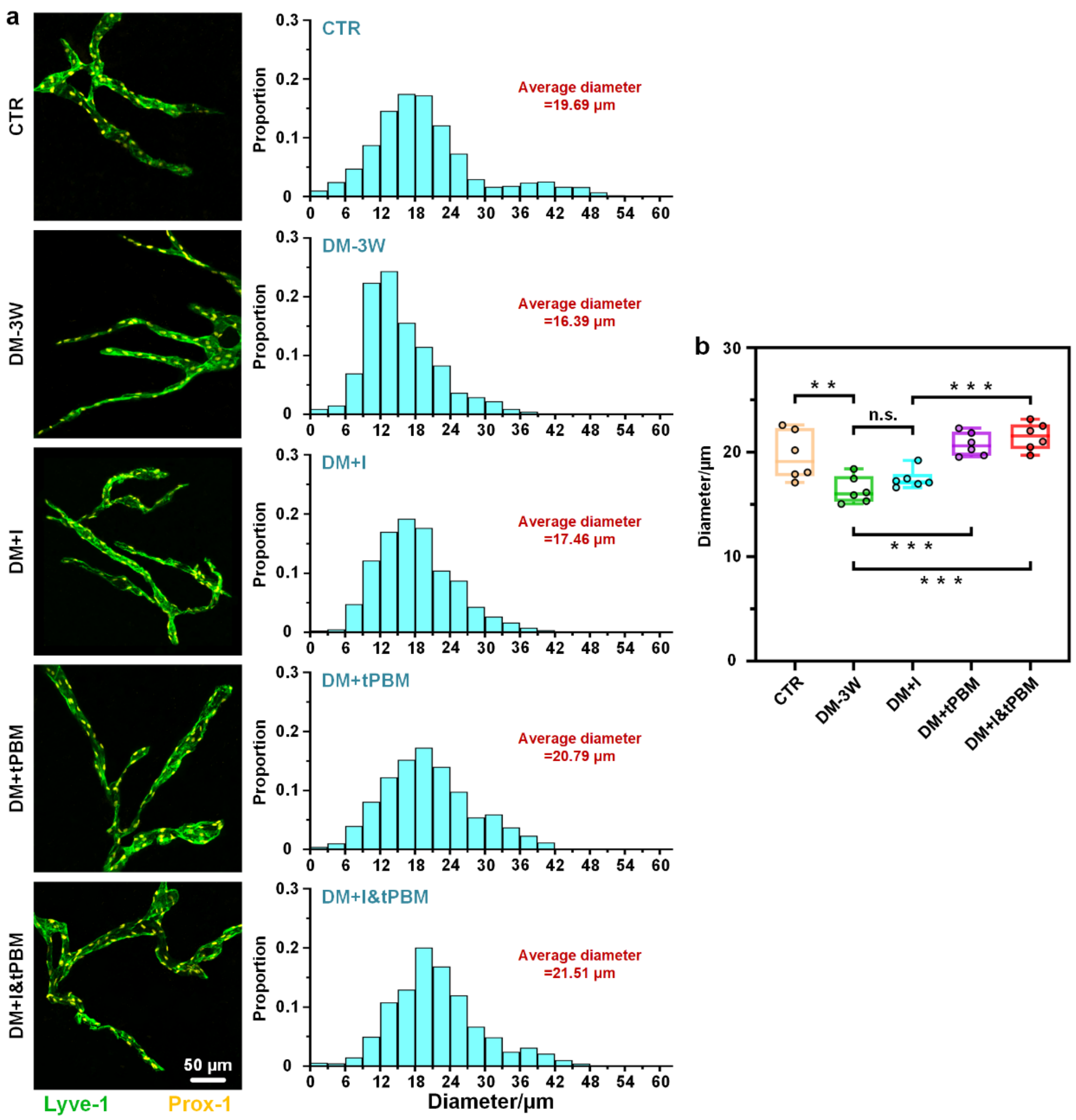

a Fluorescence images of the basal MLVs (labeled with Lyve-1, green and Prox-1, yellow) and diameter distribution histogram in the tested groups. Scale bar: 50 μm. **b** Quantitative analysis of the diameter of the MLVs in the tested groups (the box indicates the upper and lower quantiles, the thick line in the box indicates the median and whiskers indicate 2.5th and 97.5th percentiles). Data were presented as mean ± standard deviation (n=6 mice in each group), statistical significance **(b)** was assessed using one-way ANOVA, followed by Dunnett's T3 post hoc test. The statistical tests involved two-sided analyses (n.s., *, **, and *** indicate not significant, p<0.05, p<0.01 and p<0.001, respectively).

Supplementary Fig. 11 Changes of IFN-γ level in the dcLNs.

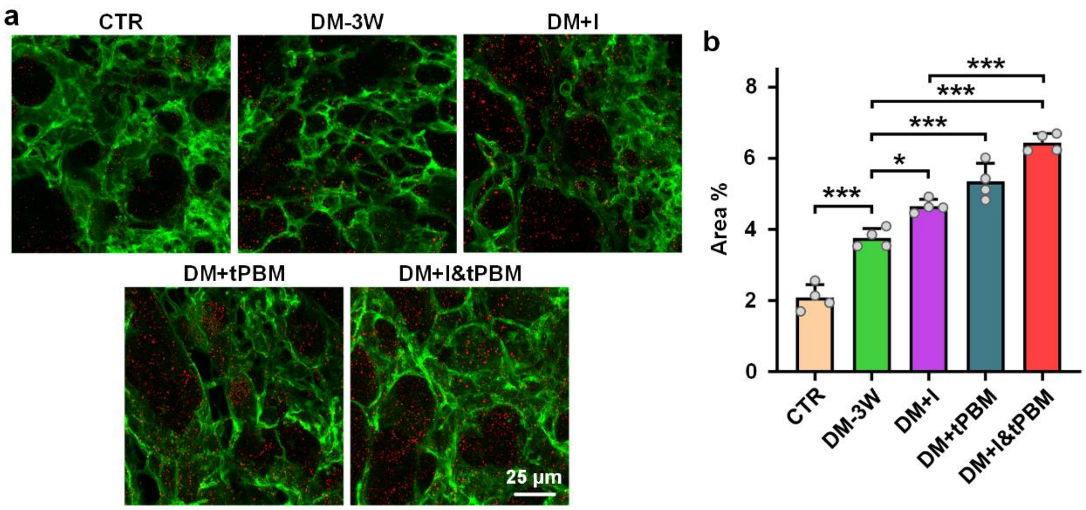

a Representative confocal images of inflammatory factor IFN-γ in the dcLNs of mice in the tested groups (green: lymphatic vessels; red: IFN-γ). Scale bar: 25 μm. **b** Quantitative analysis of IFN-γ level in the dcLNs. Data were presented as mean ± standard deviation (n=4 mice in each group), statistical significance **(b)** was assessed using one-way ANOVA, followed by Tukey's post hoc test. The statistical tests involved two-sided analyses (*, **, and *** indicate $p<0.05$, $p<0.01$, and $p<0.001$, respectively).

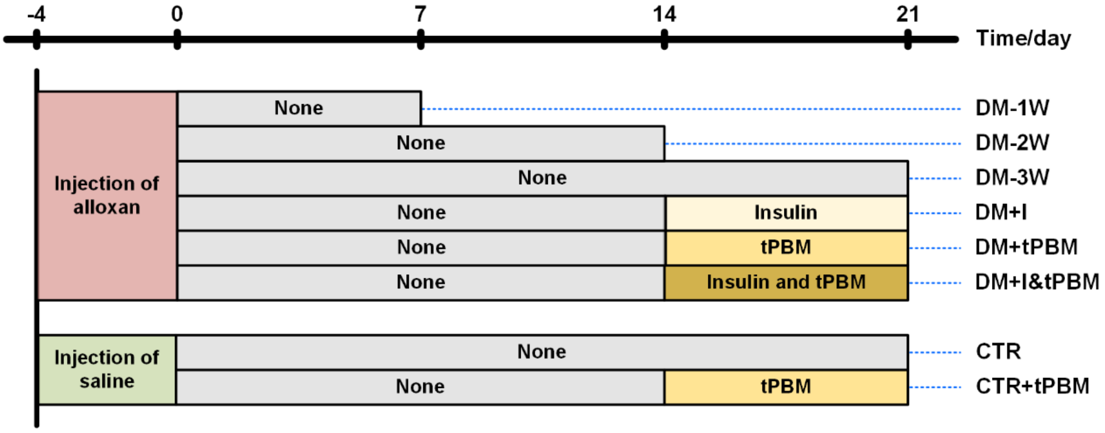

Supplementary Fig. 12 The summary figure of the tested groups and durations of treatment.

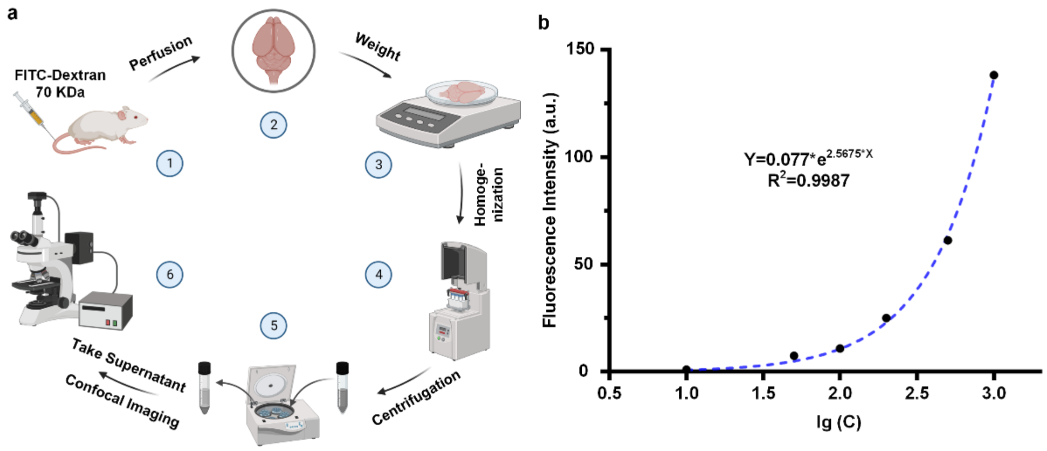

Supplementary Fig. 13 Schematic illustration of quantitative assessment of FITC-Dextran leakage. a Steps for quantitative assessment of dye leakage. **b** Standard curve between FITC-Dextran concentration and fluorescence intensity. Where *C* is the concentration of FITC-Dextran (ng/mL).

Supplementary Fig. 14 Original scanned images of the blots and gels presented in this study.

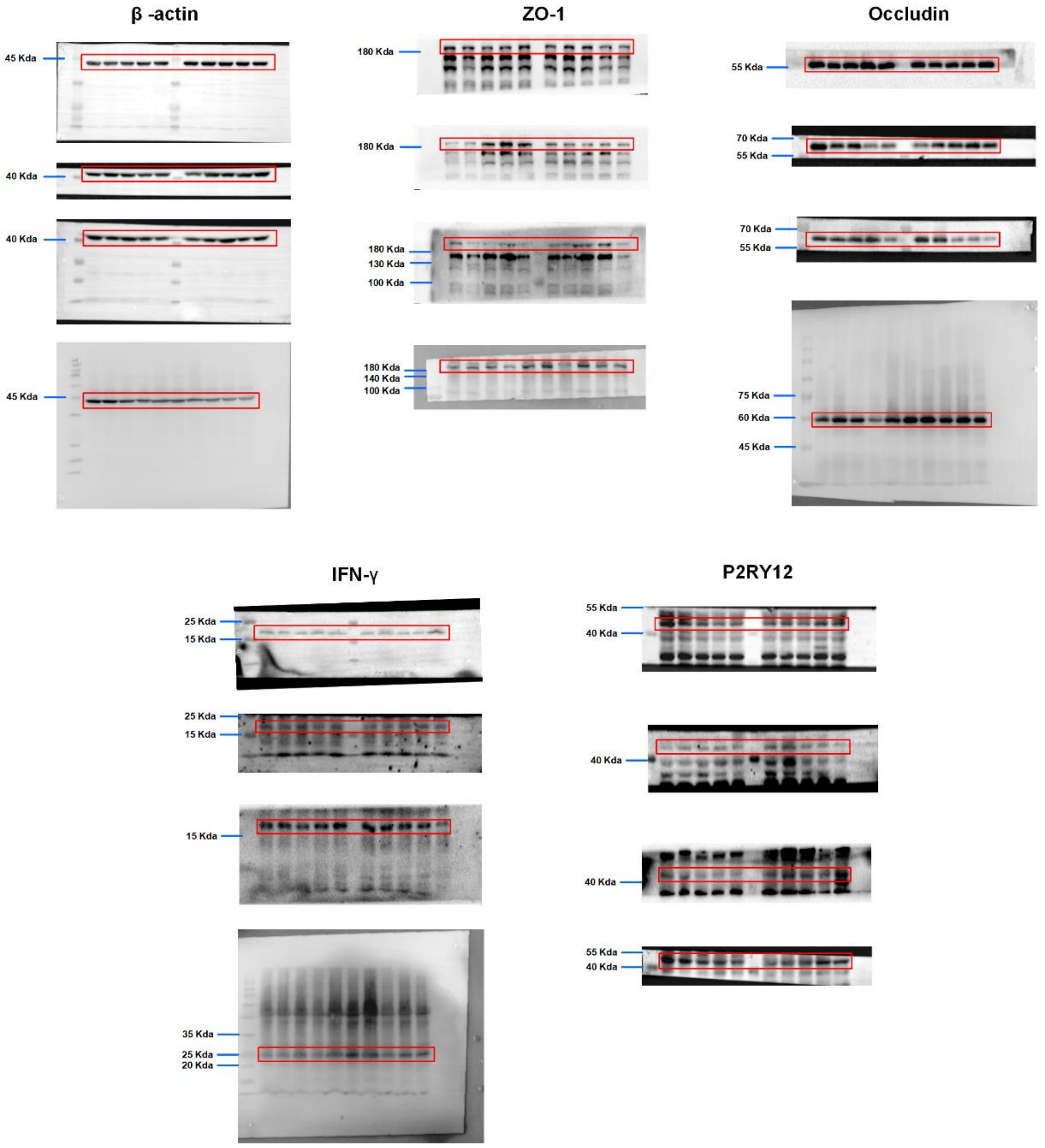

The figure panel that corresponds to each scan is labeled, and the selected region for display is marked in red box.
