# Supplementary material for: Transcranial photobiomodulation improves insulin therapy in diabetic microglial reactivity and the brain drainage system
Source: Commun Biol. 2023 Dec 8;6:1239. doi: 10.1038/s42003-023-05630-3 (PMC10709608; doi:10.1038/s42003-023-05630-3)
Supplement: Supplementary file 3 — Description of Additional Supplementary Files [file 42003_2023_5630_MOESM3_ESM.pdf]

Description of Additional Supplementary Files

File name: Supplementary Data 1
Description: The source data behind the graphs in the paper.

File name: Supplementary Movie 1
Description: Microglial reactivity to cerebrovascular injury in tested groups.
