# Supplementary material for: Transcranial photobiomodulation improves insulin therapy in diabetic microglial reactivity and the brain drainage system
Source: Commun Biol. 2023 Dec 8;6:1239. doi: 10.1038/s42003-023-05630-3 (PMC10709608; doi:10.1038/s42003-023-05630-3)
Supplement: Supplementary file 6 — Reporting Summary [file 42003_2023_5630_MOESM6_ESM.pdf]

nature portfolio

Corresponding author(s): Dan Zhu

Last updated by author(s): Nov 17, 2023

Reporting Summary

Nature Portfolio wishes to improve the reproducibility of the work that we publish. This form provides structure for consistency and transparency in reporting. For further information on Nature Portfolio policies, see our Editorial Policies and the Editorial Policy Checklist.

Statistics

For all statistical analyses, confirm that the following items are present in the figure legend, table legend, main text, or Methods section.

n/a	Confirmed	
☐	☒	The exact sample size (n) for each experimental group/condition, given as a discrete number and unit of measurement
☐	☒	A statement on whether measurements were taken from distinct samples or whether the same sample was measured repeatedly
☐	☒	The statistical test(s) used AND whether they are one- or two-sided<br>*Only common tests should be described solely by name; describe more complex techniques in the Methods section.*
☒	☐	A description of all covariates tested
☐	☒	A description of any assumptions or corrections, such as tests of normality and adjustment for multiple comparisons
☐	☒	A full description of the statistical parameters including central tendency (e.g. means) or other basic estimates (e.g. regression coefficient) AND variation (e.g. standard deviation) or associated estimates of uncertainty (e.g. confidence intervals)
☐	☒	For null hypothesis testing, the test statistic (e.g. F, t, r) with confidence intervals, effect sizes, degrees of freedom and P value noted<br>*Give P values as exact values whenever suitable.*
☒	☐	For Bayesian analysis, information on the choice of priors and Markov chain Monte Carlo settings
☒	☐	For hierarchical and complex designs, identification of the appropriate level for tests and full reporting of outcomes
☒	☐	Estimates of effect sizes (e.g. Cohen's d, Pearson's r), indicating how they were calculated

Our web collection on statistics for biologists contains articles on many of the points above.

Software and code

Policy information about availability of computer code

Data collection	The commercial softwares used in this study for data collection are: Zen 2011 SP2 (Version 8.0.0.273, Carl Zeiss GmbH), NIS-Elements AR (Version 4.20, Nikon).
Data analysis	The commercial softwares used in this study for data analysis are: Imaris (Version 7.6, Bitplane AG), SPSS (Version 26, IBM), Matlab (Version 2014a, Mathworks).<br>The open source software used in this study to analyze the data is: ImageJ (Version 1.51j8, NIH).<br>All the custom-made codes: The codes for analyzing the function of microglia and the diameter of meningeal lymphatic vessels are freely available on Github [https://github.com/HUST-LAB/microglia-MLV-analysis].

For manuscripts utilizing custom algorithms or software that are central to the research but not yet described in published literature, software must be made available to editors and reviewers. We strongly encourage code deposition in a community repository (e.g. GitHub). See the Nature Portfolio guidelines for submitting code & software for further information.

Data

Policy information about availability of data

All manuscripts must include a data availability statement. This statement should provide the following information, where applicable:

- Accession codes, unique identifiers, or web links for publicly available datasets
- A description of any restrictions on data availability
- For clinical datasets or third party data, please ensure that the statement adheres to our policy

The source data for the graphs in this study are provided in Supplementary Data 1. All other supporting data of this study are available from the corresponding author upon reasonable request.

Human research participants

Policy information about studies involving human research participants and Sex and Gender in Research.

Reporting on sex and gender	*Use the terms sex (biological attribute) and gender (shaped by social and cultural circumstances) carefully in order to avoid confusing both terms. Indicate if findings apply to only one sex or gender; describe whether sex and gender were considered in study design whether sex and/or gender was determined based on self-reporting or assigned and methods used. Provide in the source data disaggregated sex and gender data where this information has been collected, and consent has been obtained for sharing of individual-level data; provide overall numbers in this Reporting Summary. Please state if this information has not been collected. Report sex- and gender-based analyses where performed, justify reasons for lack of sex- and gender-based analysis.*
Population characteristics	*Describe the covariate-relevant population characteristics of the human research participants (e.g. age, genotypic information, past and current diagnosis and treatment categories). If you filled out the behavioural & social sciences study design questions and have nothing to add here, write "See above."*
Recruitment	*Describe how participants were recruited. Outline any potential self-selection bias or other biases that may be present and how these are likely to impact results.*
Ethics oversight	*Identify the organization(s) that approved the study protocol.*

Note that full information on the approval of the study protocol must also be provided in the manuscript.

Field-specific reporting

Please select the one below that is the best fit for your research. If you are not sure, read the appropriate sections before making your selection.

☒ Life sciences ☐ Behavioural & social sciences ☐ Ecological, evolutionary & environmental sciences

For a reference copy of the document with all sections, see nature.com/documents/nr-reporting-summary-flat.pdf

Life sciences study design

All studies must disclose on these points even when the disclosure is negative.

Sample size	Sample size was determined according to previously publications and the number of biological replicates necessary for ensuring statistical significance. Please see the figure legends for details.
Data exclusions	No data were excluded.
Replication	Experiments were repeated in at least 2 independent experiments with similar results. All experiments were reproduced to reliably support the conclusions stated in the manuscript.
Randomization	Animals were randomized into the treatment groups.
Blinding	The investigators were blinded to group allocation during data collection and analysis.

Reporting for specific materials, systems and methods

We require information from authors about some types of materials, experimental systems and methods used in many studies. Here, indicate whether each material, system or method listed is relevant to your study. If you are not sure if a list item applies to your research, read the appropriate section before selecting a response.

Materials & experimental systems

n/a	Involved in the study
☐	☒ Antibodies
☒	☐ Eukaryotic cell lines
☒	☐ Palaeontology and archaeology
☐	☒ Animals and other organisms
☒	☐ Clinical data
☒	☐ Dual use research of concern

Methods

n/a	Involved in the study
☒	☐ ChIP-seq
☒	☐ Flow cytometry
☒	☐ MRI-based neuroimaging

Antibodies

Antibodies used

Primary antibodies: anti-ZO-1 antibody (ab96587, Abcam, dilution 1:500); anti-Occludin (ab216327, Abcam, dilution 1:1000); anti-IFN-γ (16731181, Invitrogen, dilution 1:500); anti-P2RY12 (ab184411, Abcam, dilution 1:2000); Alexa Fluor 488-conjugated anti-Lyve-1 (FAB2125G, R&D Systems, dilution 1:500); anti-Prox-1 (ab101851, Abcam, dilution: 1:500); anti-Mki67 (K009725P, Solarbio®, dilution 1:500); DAPI (Invitrogen, D1306, dilution:1:1000).
Secondary antibody: Alexa Fluor 555 goat anti-rabbit IgG (H+L) (1:500, A21429, Invitrogen, dilution 1:500); Alexa Fluor 647 goat anti-rabbit IgG (H+L) (A21245, Invitrogen, dilution 1:500).

Validation

The anti-ZO-1 (ab96587, Abcam) has been validated by the manufacturer: Abcam, https://www.abcam.cn/products/primary-antibodies/zo1-tight-junction-protein-antibody-ab96587.html.
The anti-Occludin (ab216327, Abcam) has been validated by the manufacturer: Abcam, https://www.abcam.cn/products/primary-antibodies/occludin-antibody-epr20992-ab216327.html.
The anti-IFN-γ (16731181, Invitrogen) has been validated by the manufacturer: Invitrogen, https://www.thermofisher.cn/cn/zh/antibody/product/IFN-gamma-Antibody-clone-XMG1-2-Monoclonal/16-7311-81.
The anti-P2RY12 (ab184411, Abcam) has been validated by the manufacturer: Abcam, https://www.abcam.cn/products/primary-antibodies/p2y12-antibody-epr18611-ab184411.html.
The Alexa Fluor 488-conjugated anti-Lyve-1 (FAB2125G, R&D Systems) has been validated by the manufacturer: R&D Systems, https://www.rndsystems.com/cn/products/mouse-lyve-1-alexa-fluor-488-conjugated-antibody-223322_fab2125g.
The anti-Prox-1 (ab101851, Abcam) has been validated by the manufacturer: Abcam, https://www.abcam.cn/products/primary-antibodies/prox1-antibody-bsa-and-azide-free-ab101851.html.
The anti-Mki67 (K009725P, Solarbio®) has been validated by the manufacturer: Solarbio®, https://www.solarbio.com/goods.php?id=73700.
The DAPI (D1306, Invitrogen) has been validated by the manufacturer: Invitrogen, https://www.thermofisher.cn/order/catalog/product/D1306.

Animals and other research organisms

Policy information about studies involving animals; ARRIVE guidelines recommended for reporting animal research, and Sex and Gender in Research

Laboratory animals

Balb/c mice were supplied by the Wuhan University Center for Animal Experiment (Wuhan, China).
Transgenic mice expressing enhanced green fluorescent protein in microglia (CX3CR1-EGFP/+) were purchased from the Jackson Laboratory (Bar Harbor, ME, USA).

Wild animals

The study did not involve wild animals.

Reporting on sex

Both sexes mice were used in the study design.

Field-collected samples

The study did not involve samples collected from the field.

Ethics oversight

The guidelines were approved by the Institutional Animal Ethics Committee of Huazhong University of Science and Technology.

Note that full information on the approval of the study protocol must also be provided in the manuscript.
